# Dosimetric consequences of the shift towards computed tomography guided target definition and planning for breast conserving radiotherapy

**DOI:** 10.1186/1748-717X-3-6

**Published:** 2008-01-31

**Authors:** Hans Paul van der Laan, Wil V Dolsma, John H Maduro, Erik W Korevaar, Johannes A Langendijk

**Affiliations:** 1 Department of Radiation Oncology, University Medical Center Groningen/University of Groningen, Hanzeplein 1, 9700 RB Groningen, The Netherlands

## Abstract

**Background:**

The shift from conventional two-dimensional (2D) to three-dimensional (3D)-conformal target definition and dose-planning seems to have introduced volumetric as well as geometric changes. The purpose of this study was to compare coverage of computed tomography (CT)-based breast and boost planning target volumes (PTV), absolute volumes irradiated, and dose delivered to the organs at risk with conventional 2D and 3D-conformal breast conserving radiotherapy.

**Methods:**

Twenty-five patients with left-sided breast cancer were subject of CT-guided target definition and 3D-conformal dose-planning, and conventionally defined target volumes and treatment plans were reconstructed on the planning CT. Accumulated dose-distributions were calculated for the conventional and 3D-conformal dose-plans, taking into account a prescribed dose of 50 Gy for the breast plans and 16 Gy for the boost plans.

**Results:**

With conventional treatment plans, CT-based breast and boost PTVs received the intended dose in 78% and 32% of the patients, respectively, and smaller volumes received the prescribed breast and boost doses compared with 3D-conformal dose-planning. The mean lung dose, the volume of the lungs receiving > 20 Gy, the mean heart dose, and volume of the heart receiving > 30 Gy were significantly less with conventional treatment plans. Specific areas within the breast and boost PTVs systematically received a lower than intended dose with conventional treatment plans.

**Conclusion:**

The shift towards CT-guided target definition and planning as the golden standard for breast conserving radiotherapy has resulted in improved target coverage at the cost of larger irradiated volumes and an increased dose delivered to organs at risk. Tissue is now included into the breast and boost target volumes that was never explicitly defined or included with conventional treatment. Therefore, a coherent definition of the breast and boost target volumes is needed, based on clinical data confirming tumour control probability and normal tissue complication probability with the use of 3D-conformal radiotherapy.

## Background

Ever since the early days of breast cancer radiotherapy, irradiation was performed by means of tangential beams directed to treat the whole breast or chest wall [1]. With the use of tangential beams, non-target thoracic structures were avoided as much as possible. To ensure that all breast parenchyma was included into the target volume, one relied upon visible or palpable anatomy as assessed by physical examination and/or fluoroscopy [2]. Standard field borders were usually placed within a certain range outside the palpable breast, while field projections and collimator angles were verified and adapted by means of radiographic examination. To enable computed dose calculation and optimisation of wedge-fractions, one or more body-outline contours were provided on which dose-planning, with or without lung-density correction, was performed [3]. However, the breast clinical target volume (CTV), i.e. the glandular breast tissue, was never explicitly defined. Currently, breast cancer radiotherapy has gradually shifted towards computed tomography (CT)-guided treatment planning. This enabled the application of new techniques such as three-dimensional (3D)-conformal radiotherapy (3D-CRT) and intensity modulated radiotherapy (IMRT) [4,5]. With these techniques, an accurate delineation of the target volume is critical because its size and shape directly affects the amount of normal tissue irradiated. However, with regard to the definition of the breast CTV, there is still no general consensus, and target volume delineation is subject to a large interobserver variability [6,7]. This may be explained by the fact that it can be difficult to distinguish the glandular breast tissue from the surrounding fatty tissue. In an effort to solve this problem, the palpable breast is often marked with a radiopaque wire during the CT scan [6]. The breast CTV is then defined within the CT images, guided by this radiopaque wire. Subsequently, a planning target volume (PTV) can be defined and 3D-conformal breast beams can be constructed. It appeared that large discrepancies exist between a CT-guided beam set-up and beams defined during the conventional process of direct simulation [8].

The introduction of CT-guided treatment planning also seems to have influenced the way the lumpectomy cavity with corresponding CTV and PTV are defined [9]. Nowadays, surgical clips, hematoma, seroma and other surgical changes are used to define the target volume in 3D, while in the conventional setting, information was limited to the location of the scar and, when available, the position of surgical clips.

Although several investigators drew attention to the volumetric and geometric changes introduced with CT-guided treatment planning in breast conserving radiotherapy, the dosimetric consequences, i.e. target coverage and dose delivered to normal tissues, have not been clearly assessed. Therefore, the purpose of this study was to compare coverage of CT-based breast and boost planning target volumes (PTV), absolute volumes irradiated, and dose delivered to the organs at risk with conventional treatment plans and 3D-conformal breast conserving radiotherapy.

## Methods

### Patients and CT scanning

Twenty-five patients with early-stage left-sided breast cancer that underwent radiotherapy after breast-conserving surgery were included in this study. A planning CT scan in treatment position was made for each patient. Before the CT scan, skin marks were placed to locate the boost-volume isocenter and enable patient repositioning during treatment. Radiopaque wires and markers were placed to locate palpable breasts, scars, and skin marks on the CT images. In addition, markers were placed to represent the conventional field borders (i.e. a mid-sternal marker, representing the medial field border, and a marker placed 20–30 mm dorsally from the lateral palpable breast representing the lateral field border). The cranial and caudal field borders were marked 15 mm beyond the palpable breast. Patients were scanned with CT from the level of the larynx to the level of the upper abdomen, including both lungs, with a scan thickness and index of 5 mm. The CT data for all patients were transferred to the Helax-TMS 3D treatment planning system, version 6.1B (Nucletron, Veenendaal, The Netherlands). All patients provided informed consent before starting therapy, and the ethics committee at the University Medical Center Groningen approved the procedures followed.

### Reconstruction of conventional treatment plans

The markers representing the conventional field borders were used to construct two opposing tangential beams by means of virtual simulation, similar to the conventional procedure by direct simulation as performed in the past at our department. Wedge fractions were defined by evaluating dose distributions limited to a slice situated in the centre of the breast, and slices at 50 mm superior and inferior to this central slice.

To enable definition of a conventional boost PTV (PTV_CON_), a body-outline contour of the slice containing the boost-volume isocenter was derived from the CT data set. All density information was erased. The body-outline contour only contained the boost-volume isocenter, a two dimensional (2D) reconstruction of all surgical clips, and the marked location of the scar. On the basis of the position of the clips and the available pre-operative information, the assumed lumpectomy cavity was defined within the 2D body-outline contour. Subsequently, the conventional boost CTV (CTV_CON_) and the boost PTV_CON _were created by adding margins of 10 mm and 5 mm, respectively. The resulting boost PTV_CON _was then transferred into the CT data-set. The field length of the boost beams was prescribed on the basis of the surgical clips, as visualised by means of digitally reconstructed radiographs. The conventional boost plan consisted of three equally weighted photon beams with manually optimised gantry angles. Beam widths and wedge fractions were selected in such a way that the 95%-isodose closely encompassed the boost PTV_CON _in the boost central slice. Dose distributions in slices other than the boost central slice were not evaluated and no additional shielding was used. For all beams 6-MV photons were used, and an energy fluence based pencil beam algorithm was used for all dose calculations.

Eventually, a cumulative dose plan was calculated, taking into account 50 Gy for the breast plan and an additional 16 Gy for the boost plan.

### CT-guided definition of target volumes and organs at risk

The breast CT-based CTV (CTV_CT_) included the glandular breast tissue of the ipsilateral breast. In practice, the breast CTV_CT _was delineated within the extent of the radiopaque wires marking the palpable breast. The breast CTV_CT _did not extend into the pectoralis major or the ribs and did not include the skin. The breast CT-based PTV (PTV_CT_) was generated by adding a 3D-margin of 5 mm around the breast CTV_CT_. Definition of the lumpectomy cavity was guided by the position of the surgical clips and pre-operative information, but also by hematoma, seroma, and/or other surgery-induced changes, that were considered to be part of the lumpectomy cavity. The boost CTV_CT _was generated by adding a 3D-margin of 10 mm around the lumpectomy cavity. The boost PTV_CT _was generated accordingly by adding an additional margin of 5 mm. Both breast and boost PTV_CT _were restricted to 5 mm within the skin surface. The heart was contoured to the level of the pulmonary trunk superiorly, including the pericardium, excluding the major vessels. Both lungs were contoured as a single organ at risk with the automatic contouring tool of the Helax-TMS planning system, and the right breast was contoured as an organ at risk similar to the left breast CTV_CT_.

### 3D-conformal treatment planning

Conformal to the breast PTV_CT_, two opposing tangential beams were constructed. With the use of beam's-eye-view projections, gantry angles were determined to achieve maximum avoidance of the heart, ipsilateral lung and right breast. Shielding was adapted with use of a multileaf collimator (MLC). Wedges and/or a maximum of three MLC segments were added by means of forward planning to obtain a homogeneous dose distribution. Subsequently, a boost plan was created conformal to the boost PTV_CT_. It consisted of three equally weighted photon beams with gantry angles identical to those that were used with the conventional boost plan. Wedges and MLC shielding were applied in such a way that the 95%-isodose closely encompassed the boost PTV_CT _in three dimensions, and a uniform dose distribution was obtained. Eventually, a cumulative dose plan was calculated incorporating both the 3D-conformal breast and boost plan, taking into account 50 Gy for the breast plan and an additional 16 Gy for the boost plan.

### Analyses of target coverage and normal tissue dose

Target coverage was determined for both the conventional and 3D-conformal dose plans by evaluating the relative volumes of the breast PTV_CT _and the boost PTV_CT _receiving at least 95% of the prescribed dose (i.e. the CT-guided PTVs were regarded as the golden standard). For each of the cumulative dose plans, the total volume and the volume outside the CT-based PTVs receiving at least 95% of the prescribed breast and boost doses were determined. In addition, the relative volumes of the heart receiving ≥ 30 Gy (V30), the mean heart dose, the relative total volume of both lungs receiving ≥ 20 Gy (V20), the mean lung dose, the relative volume of the right breast receiving ≥ 10 Gy (V10) and the right breast mean dose were derived from the dose-volume histograms (DVH).

### Statistical analysis

For comparison of the DVH parameters of the cumulative dose plans, the mean values were analysed with the Wilcoxon signed ranks test or the paired-samples t-test on statistical significance whenever appropriate. All tests were two-tailed, and differences were considered statistically significant at *p *≤ 0.05.

## Results

### PTV_CT _coverage and absolute volumes irradiated

With conventional breast beams, coverage of the breast PTV_CT _was adequate in 72% of the patients (in these patients, ≥ 95% of the prescribed breast dose was delivered to ≥ 95% of the breast PTV_CT_). With 3D-CRT, coverage of the breast PTV_CT _was adequate for all patients (Table 1). The volume outside the breast PTV_CT _that received ≥ 95% of the prescribed breast dose was significantly smaller when conventional breast beams were used (427 cm^3 ^vs. 529 cm^3 ^with 3D-CRT).

**Table 1:** Target coverage and irradiated volumes

	Cumulative dose plan CT-based	Cumulative dose plan Conventional	*p*-values
			
*Target coverage *(%)			
Breast PTV_CT _≥ 95%	99.2 (97.7 – 100.0)	95.3 (84.5 – 100.0)	< 0.001
Boost PTV_CT _≥ 95%	99.6 (98.0 – 100.0)	90.1 (70.3 – 100.0)	< 0.001
*Irradiated volumes *(cm^3^)			
Volume ≥ 95% * 50 Gy	1276 (461 – 2239)	1142 (518 – 1968)	0.01
Volume ≥ 95% * 66 Gy	241 (105 – 491)	187 (84 – 376)	< 0.001
*Excess volumes *(cm^3^)			
≥ 95% * 50 Gy outside breast PTV_CT_	529 (257–1114)	427 (143 – 857)	0.02
≥ 95% * 66 Gy outside boost PTV_CT_	124 (65 – 260)	82 (31 – 144)	< 0.001

PTV: planning target volume; PTV_CT_: computed tomography (CT)-based PTV.

Data presented as mean values, with ranges in parentheses

With conventional boost beams, coverage of the boost PTV_CT _was adequate in only 32% of the patients, while coverage was adequate for all patients with 3D-CRT. The volume outside the boost PTV_CT _that received ≥ 95% of the prescribed boost dose was significantly less when conventional beams were used (82 cm^3 ^vs. 124 cm^3 ^with 3D-CRT).

### Organs at Risk

The mean heart dose and the heart V5–V30 were significantly larger with the use of 3D-CRT (Table 2). Similar results were observed with regard to the mean lung dose and the lung V5–V30. The right breast mean dose and right breast V5–V30 were minimal and similar for the 3D-CRT and conventional cumulative dose plans.

**Table 2:** Mean dose and percentage of volume of heart, lungs and right breast irradiated

Organs at Risk	CT-based cumulative dose plan	Conventional cumulative dose plan	*p*-values
			
*Heart*			
Volume ≥ 30 Gy (%)	3.6 (0.0 – 10.9)	1.4 (0.0 – 7.9)	< 0.001
Volume ≥ 20 Gy (%)	5.1 (0.0 – 14.0)	1.9 (0.0 – 9.5)	< 0.001
Volume ≥ 10 Gy (%)	9.0 (0.0 – 22.7)	4.9 (0.0 – 18.0)	< 0.001
Volume ≥ 5 Gy (%)	27.4 (9.5 – 57.4)	20.5 (0.0 – 47.7)	< 0.001
Mean dose (Gy)	5.5 (2.5 – 10.8)	4.0 (1.4 – 9.0)	< 0.001
*Lungs*			
Volume ≥ 30 Gy (%)	4.7 (1.2 – 10.1)	3.5 (0.0 – 9.1)	0.014
Volume ≥ 20 Gy (%)	5.6 (1.7 – 11.1)	4.2 (0.2 – 10.2)	0.006
Volume ≥ 10 Gy (%)	8.2 (2.6 – 14.2)	6.7 (2.5 – 14.1)	0.010
Volume ≥ 5 Gy (%)	16.6 (5.2 – 28.1)	14.7 (7.3 – 26.6)	0.006
Mean dose (Gy)	4.7 (2.0 – 8.4)	4.0 (2.1 – 7.4)	0.011
*Right breast*			
Volume ≥ 30 Gy (%)	0.1 (0.0 – 0.9)	0.0 (0.0 – 0.0)	ns
Volume ≥ 20 Gy (%)	0.2 (0.0 – 2.9)	0.0 (0.0 – 0.0)	ns
Volume ≥ 10 Gy (%)	0.3 (0.0 – 4.7)	0.0 (0.0 – 0.2)	ns
Volume ≥ 5 Gy (%)	0.8 (0.0 – 7.8)	0.2 (0.0 – 1.8)	ns
Mean dose (Gy)	0.9 (0.3 – 2.7)	0.9 (0.5 – 1.7)	ns

Data presented as mean values, with ranges in parentheses

### Conventional field borders in relation to PTV_CT_

**Figure 1:**
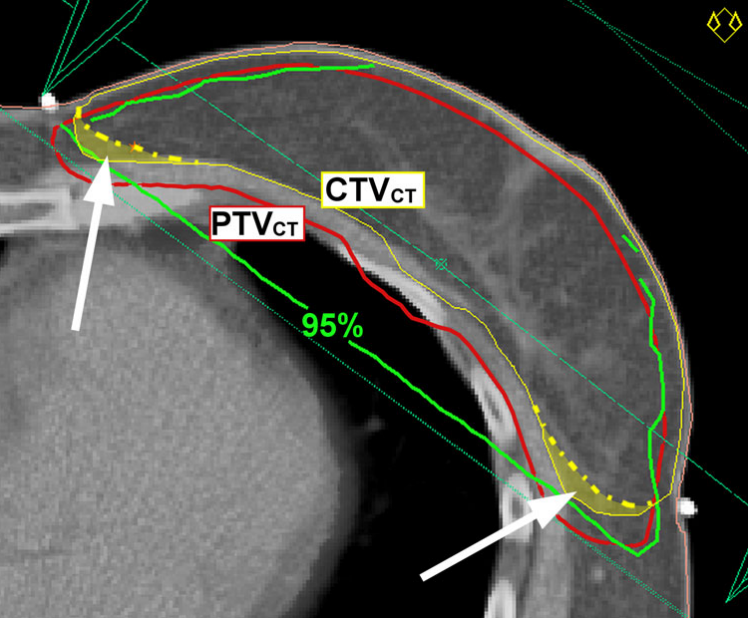
**Dose-distribution conventional breast beams relative to CT-based breast target volumes**. Representation of 95%-isodose (green) resulting from conventional breast beams and computed tomography (CT)-based clinical target volume (CTV) and planning target volume (PTV). Note the areas of PTV (red) not covered by 95%-isodose when conventional beams are used. Under-dosage of PTV is caused by including additional tissue (marked yellow-wash areas) into the CTV.

Conventional breast beams resulted in poor coverage of the medio-dorsal and latero-dorsal areas of the breast PTV_CT _in the majority of the patients (Fig. 1). Particularly the latero-dorsal areas of the breast PTV_CT _significantly extended beyond conventional field borders (Table 3).

**Table 3:** PTV volumes and dimensions

	CT-based target definition	Conventional target definition	*p*-values
			
*Absolute volumes *(cm^3^)			
Breast PTV	753 (209 – 1548)	-	-
Boost PTV	117 (40 – 243)	-	-
*Breast PTV_CT _beyond conventional field borders *(cm)			
Medial	0.03 (-1.5 – 1.1)		ns
Lateral	0.37 (-1.1 – 1.2)		0.01
*Dimensions boost fields and PTVs *(cm)			
Field length	7.9 (5.4 – 10.4)	6.7 (4.5 – 8.5)	< 0.001
PTV_CT _extending beyond PTV_CON_			
right	0.18 (-0.6 – 1.2)		0.03
left	0.36 (-0.4 – 1.7)		0.01
ventral	0.22 (-1.7 – 1.7)		ns
dorsal	0.23 (-0.7 – 2.5)		ns

PTV: planning target volume; PTV_CT_: computed tomography (CT)-based PTV; PTV_CON_: conventional PTV. Data presented as mean values, with ranges in parentheses.

**Figure 2:**
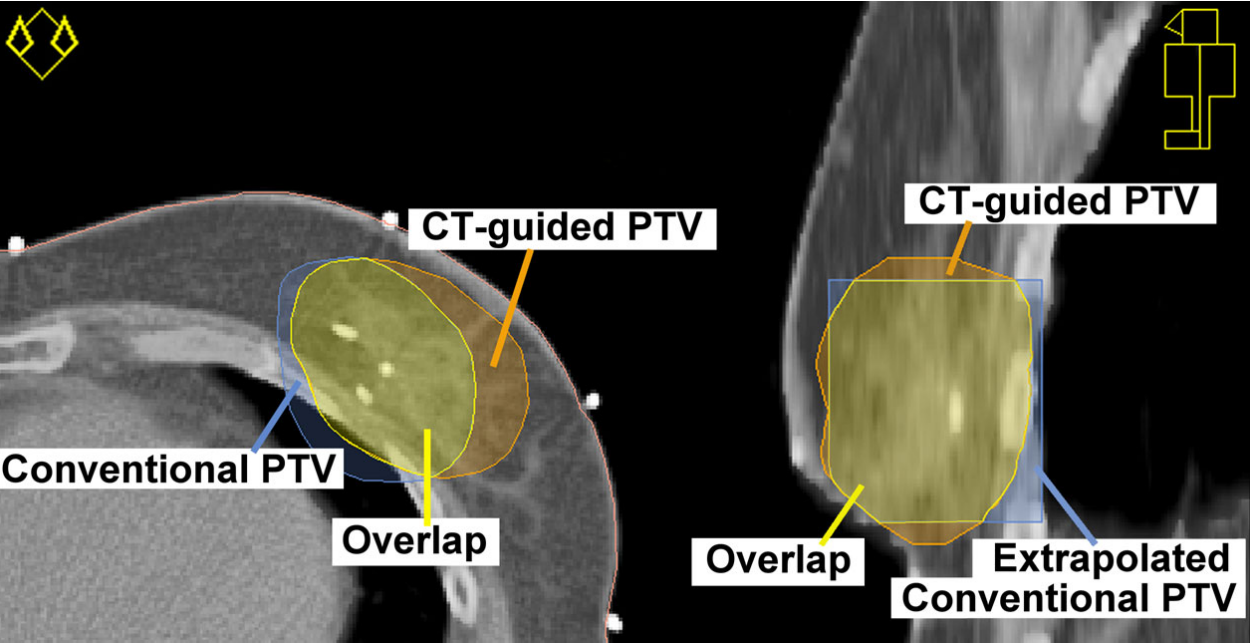
**Conventional and CT-based boost planning target volume**. Transversal (left) and sagittal (right) cross-sections of conventional and computed tomography (CT)-based boost planning target volume (PTV).

The boost PTV_CT _generally extended beyond the boost PTV_CON _in the medial and lateral directions (Fig. 2) and Table 3). In the ventral and dorsal directions, the PTV_CT _and PTV_CON _were mutually divergent in most cases, however no significant differences were found. The cranial and caudal borders of the 3D-CRT boost beams extended beyond the conventional boost beams in the majority of patients.

## Discussion

On the basis of the current analysis we conclude that CT-guided target definition and planning for breast conserving radiotherapy results in improved target coverage at the cost of an increased dose delivered to organs at risk. It seems that when CT densities are used to define the breast CTV, tissue is included that would not have been specifically targeted with conventional breast beams. However, it is uncertain whether or not the additional included tissue is really breast tissue at risk.

The dosimetric results with 3D-CRT strongly depend on institutional guidelines used for delineation of the breast target volumes. Various methods have been used in the past to delineate the breast CTV: anatomic references have been used as a guide [10,11], but also radiopaque wires marking the palpable breast [6,12]. In some studies, a conventional beam set-up was used even when CT data were available [13,14]. In these studies, CT data were used for dose calculation and evaluation of the dose to organs at risk, while the breast CTV was not explicitly defined. In addition, no margins for position uncertainties or penumbra were specified, while in other studies, a 5–7 mm margin for position uncertainties was used together with a margin for penumbra [12,15]. This illustrates that consensus is needed on how the breast target volumes should be defined within the CT images. The delineation method used in the present study, resulted in relatively consistent results because the information used was threefold: 1) palpable breast tissue marked by a radiopaque wire; 2) glandular breast tissue as visible in the CT images; and 3) the use of anatomic references. Therefore, we consider this method to be the current golden standard for CT-guided target definition in breast conserving RT.

Patient selection was started more than one year after the introduction of CT-guided target definition and planning as standard procedure for breast conserving RT at our institution. Therefore, all involved physicians had at least one year of experience, while there were regular interobserver consultations to discuss the delineation of the target volumes. In this way, the effect of a learning curve was eliminated as much as possible.

In the present study, the tangential beams of the conventional and 3D-CRT plans were not adjusted when they included more normal tissue than expected. However, in our clinical practice, the gantry angles of the tangential beams are adjusted when the contralateral breast is partially included or when the central lung distance exceeds 30 mm. In some patients, avoidance of the contralateral breast is not possible without a significant increase of the dose delivered to the lungs. In these cases, inadequate coverage of the medial and lateral aspects of the breast PTV is accepted as long as adequate coverage of the boost PTV is maintained.

The position of the conventional breast beams was evaluated in relation to the breast PTV_CT_. Although 3D-CRT field sizes were predominantly larger than conventional field sizes, in some cases the resulting 3D-CRT fields were actually smaller than the conventional fields. As shown in Table 3, the medial aspect of the breast PTV_CT _was in some cases positioned as far as 1.5 cm within the conventional field borders, while the lateral aspect of the breast PTV_CT _was in some cases positioned as far as 1.1 cm within the conventional field borders.

In the present study, CT-guided target definition and planning resulted in larger boost PTVs that were inadequately covered in 68% of the cases when conventional boost beams were used. Although the volume increase can be partly explained by the additional density information provided by CT, it also appeared that with CT-guided planning, the margins for penumbra needed in the cranial and caudal directions could measure up to 10 mm. We conclude that margins for position uncertainties and penumbra were not fully taken into account when the field lengths were prescribed for the conventional boost beams.

In most cases, the boost PTV_CT _extended beyond PTV_CON_, resulting in larger boost volumes with 3D-CRT. In some patients, however, the CT-based lumpectomy cavity was defined to (marginally) exclude one or more of the surgical clips when these appeared remote from the lumpectomy cavity. In these patients, the PTV_CON _extended beyond the PTV_CT _in one or more directions.

Equally weighted boost beams were used in the current study. In our clinical practice, the boost-beam weights are optimized for each individual patient. However, the two treatment methods had different optimum boost-beam weights. For methodological reasons, optimisation of the boost-beam weights was not performed separately for the two treatments.

While photon beams were used for boost irradiation in the present study, others reported on the dosimetric results with an electron boost. It was demonstrated by Benda et al. [16] that target coverage with electron beams, determined without the use of CT data, resulted in very poor target coverage (with on average only 51% of the CT-guided boost PTV receiving 90% or more of the prescribed dose). It is likely that such inadequate coverage of the boost volume has also been the case in the "boost vs. no boost" trial [17,18]. This trial showed that an additional boost dose of 16 Gy, delivered with the use of conventional photon or electron techniques, significantly reduced the risk of a local recurrence. Because it has been demonstrated that in most cases, local recurrences occur close to the primary tumour site [19], it may be possible that CT-guided target definition in conjunction with 3D-conformal dose-planning will further reduce the risk of local recurrence as the dose distribution to the lumpectomy cavity is more adequate.

CT-guided target definition and planning resulted in higher doses delivered to the heart and lungs because larger tangential beams were needed to include the breast PTV_CT_. The largest increase was observed with the heart V30. Although the absolute increase in normal tissue dose seems to be relatively small, clinical consequences can never be ruled out and attempts should always be made to minimise the dose delivered to organs at risk. A number of studies pointed out that patients who received partial irradiation of the heart had an increased risk of dying from cardiac disease [20-22]. In these studies, conventional radiotherapy techniques were used. The present study demonstrates that the introduction of CT-guided target definition and planning may result in an increase of the dose delivered to the heart in some cases. Other authors already reported on restricting the 3D-CRT field edges in the vicinity of the heart and the application of cardiac shielding to reduce the heart dose [23]. We also tested this method at our institute in three patients that had upper-quadrant tumour sites. It appeared that the heart V30 could be reduced to 0% at the cost of reduced coverage of the breast PTV_CT _(Table 4). Although the use of cardiac shielding was not specifically analysed as a part of the current study, it could be regarded as a first and rather safe step towards partial breast irradiation in selected patients who have early-stage disease at locations remote from the heart. In this way, it may be possible to reduce the heart dose with 3D-CRT even below the levels resulting from conventional treatment. A large randomized trial would be necessary to determine tumour control probability and normal tissue complication probability with the different uses of 3D-conformal techniques in breast conserving radiotherapy.

**Table 4:** Heart dose and target coverage with and without conformal shielding of the heart

	Heart				Breast PTV_CT_	
	Mean dose (Gy)		Volume ≥ 30 Gy (%)		Volume receiving ≥ 95% of prescribed dose (%)	

	3D-CRT	3D-CRT with shielded heart	3D-CRT	3D-CRT with shielded heart	3D-CRT	3D-CRT with shielded heart

*Patient*						
1	4.8	2.0	5.1	0.0	98.9	93.8
2	2.9	2.1	1.4	0.0	98.9	97.5
3	3.4	2.0	2.5	0.0	99.1	90.3

Dosimetric results with and without deliberate multileaf collimator shielding of the heart in the tangential breast beams. Results based on cumulative dose plans (breast plan 50 Gy + boost plan 16 Gy). Patients had upper-quadrant tumor sites. Boost PTV_CT _target coverage was not compromised.

3D-CRT: three-dimensional conformal radiation therapy; PTV: planning target volume; PTV_CT_: computed tomography (CT)-based PTV.

## Conclusion

The shift towards CT-guided target definition and planning as the golden standard for breast conserving radiotherapy has resulted in improved target coverage at the cost of larger irradiated volumes and an increased dose delivered to organs at risk. Tissue is now included into the breast and boost target volumes that was never explicitly defined or included with conventional treatment. Therefore, a coherent definition of the breast and boost target volumes is needed, based on clinical data confirming tumour control probability and normal tissue complication probability with the use of 3D-conformal radiotherapy.

## Competing interests

The authors declare that they have no competing interests.

## Authors' contributions

HPvdL designed and coordinated the study, performed dose-planning and dose-calculation, performed the data collection and analysis and drafted the manuscript. WVD participated in the design of the study, performed the definition of the conventional 2D target volumes and authorised virtual simulation of conventional treatment plans. JHM participated in the design of the study and assisted in the definition of conventional 2D target volumes. EWK participated in the design of the study and helped to draft the manuscript. JAL conceived of the study, participated in its design and coordination and helped to draft the manuscript. All authors read and approved the final manuscript.
